# Supplementary material for: Mangrove Ecosystems as Reservoirs of Antibiotic Resistance Genes: A Narrative Review
Source: Antibiotics (Basel). 2025 Oct 14;14(10):1022. doi: 10.3390/antibiotics14101022 (PMC12561704; doi:10.3390/antibiotics14101022)
Supplement: Supplementary file 1 [file antibiotics-14-01022-s001.zip › antibiotics-3895882-supplementary/antibiotics-3895882-Diagram S1.pdf]

Supplementary Flow Diagram S1. Schematic overview of the narrative review workflow and key findings

A. Workflow of Study Selection

1. **Literature search**
    - Databases: PubMed, Scopus, Web of Science, Google Scholar
    - Keywords: “antibiotic resistance genes”, “ARB”, “resistome”, “mangrove”, etc.
    - Timeframe: 2008 – Sept 2024; 2025 in-press/early online included
2. **Screening**
    - Titles/abstracts screened by lead author
    - Full texts reviewed for eligibility
    - Co-author verification for consistency
    - Manual removal of duplicates
3. **Inclusion criteria**
    - Primary data on ARGs/ARB in mangrove sediments, water, or biota
    - Molecular, culture-based, or metagenomic methods
    - Published in English with sufficient methodological detail
4. **Data extraction**
    - Summarized narratively: study location, sample type, detection method, ARG types, abundance, environmental factors
5. **Quality assessment**
    - Narratively evaluated: clarity of objectives, sampling, detection methods, data analysis
    - Lead author with co-author verification; no formal scoring
6. **Data synthesis**
    - Narrative synthesis highlighting trends in ARG distribution, diversity, microbial communities, environmental drivers

B. Key Findings in Mangrove Ecosystems

- **Sample types:** sediments, water, associated biota
- **ARGs:** tetracyclines, sulfonamides, β-lactams, multidrug, aminoglycosides, macrolides, quinolones
- **Microbial carriers:** *Proteobacteria*, *Firmicutes, Bacteroidetes*; opportunistic pathogens like *Enterococcus, Vibrio, Pseudomonas*
- **Environmental drivers:** aquaculture proximity, urban runoff, sediment type, organic matter, salinity
- **Patterns:** Seasonal fluctuations, higher abundance near human activity, co-occurrence with mobile genetic elements

A. Workflow of Study Selection

1. Literature search
- Databases: PubMed, Scopus, Web of Science, Google Scholar
- Keywords: “antibiotic resistance genes”, “ARB”, “resistome”, “mangrove”, etc.
- Timeframe: 2008 – Sept 2024; 2025 in-press/early online included

2. Screening
- Titles/abstracts screened by lead author
- Full texts reviewed for eligibility
- Co-author verification for consistency
- Manual removal of duplicates

3. Inclusion criteria
- Primary data on ARGs/ARB in mangrove sediments, water, or biota
- Molecular, culture-based, or metagenomic methods
- Published in English with sufficient methodological detail

4. Data extraction
- Summarized narratively: study location, sample type, detection method, ARG types, abundance, environmental factors

5. Quality assessment
- Narratively evaluated: clarity of objectives, sampling, detection methods, data analysis
- Lead author with co-author verification; no formal scoring

6. Data synthesis
- Narrative synthesis highlighting trends in ARG distribution, diversity, microbial communities, environmental drivers

B. Key Findings in Mangrove Ecosystems

Sample types
- sediments
- water
- associated biota

ARGs
- tetracyclines
- sulfonamides
- β-lactams
- aminoglycosides
- macrolides
- quinolones

Microbial carriers
- *Proteobacteria*
- *Firmicutes*
- *Bacteroidetes*
- opportunistic pathogens like *Enterococcus, Vibrio, Pseudomonas*

Environmental drivers
- aquaculture proximity
- urban runoff
- sediment type
- organic matter
- salinity

Patterns:
- Seasonal fluctuations
- higher abundance near human activity
- co-occurrence with mobile genetic elements

Key Findings: Mangroves contain diverse ARGs (tetracyclines, β-lactams, aminoglycosides, etc.) carried by bacteria including Proteobacteria and opportunistic pathogens. ARG abundance is influenced by aquaculture, urban runoff, sediment, organic matter, and salinity, showing seasonal fluctuations and higher levels near human activity, often linked to mobile genetic elements.
