# Supplementary material for: Mangrove Ecosystems as Reservoirs of Antibiotic Resistance Genes: A Narrative Review
Source: Antibiotics (Basel). 2025 Oct 14;14(10):1022. doi: 10.3390/antibiotics14101022 (PMC12561704; doi:10.3390/antibiotics14101022)
Supplement: Supplementary file 1 [file antibiotics-14-01022-s001.zip › antibiotics-3895882-supplementary/antibiotics-3895882-supplementary.pdf]

Supplementary Table S1. Summary of Included Studies on ARGs in Mangrove Ecosystems

Study	Country / Region	Sample Type	Detection Method	ARGs Detected	Key Findings	Reference
Jiang et al., 2021	China (Hainan)	Sediment	qPCR	*tetA, tetM, sul1*	ARG abundance higher near aquaculture	[11]
Palacios et al., 2021	Mexico	Water	Metagenomics	*mdt, acr*	ARG detectable in urban-influenced mangroves	[1]
Zhao et al., 2023	South China	Sediment	Metagenomics	*sul2, bla_CTX-M*	Higher ARGs in fine, organic-rich sediments	[15]
Imchen et al., 2019	India	Sediment	16S + qPCR	*tetM, bla_TEM*	Human-impacted mangrove sites enriched in ARGs	[14]
Zhang et al., 2009	China	Water & Sediment	Metagenomics	*tetA, sul1, bla_CTX-M, mdt*	High prevalence of mobile genetic elements	[13]
Hendriksen et al., 2019	Global	Sediment	Metagenomics	Various ARGs	Comparative global sewage resistomes	[4]
Sresung et al., 2024	Thailand	Water	qPCR	*sul1, sul2*	ARGs associated with urban runoff	[6]
Yaikhan et al., 2024	Thailand	Sediment	PCR/qPCR	*blaTEM, blaSHV*	β-lactam resistance in aquaculture-influenced sediments	[7]
Hinthong et al., 2024	Thailand	Water	qPCR	*blaCTX-M*	ARGs in water adjacent to aquaculture	[9]
Intahphuak et al., 2021	Thailand	Sediment	qPCR	*ermB, mefA*	Macrolide resistance in human-impacted sites	[10]
Liu et al., 2023	China	Sediment	Metagenomics	*qnrS, qnrB*	Quinolone resistance in coastal sediments	[2]
Cabello et al., 2013	Chile	Sediment	Culture-based + qPCR	*mdtK, acrB*	ARGs prevalent in aquaculture sediments	[16]
Zhang et al., 2020	China	Water	Metagenomics	*qnrS, qnrB*	Quinolone resistance detected in mangroves	[5]

Notes / Footnotes:

1. **ARG abundance:** Number of copies per gram sediment or per mL water, as reported in original studies
2. **Key Findings:** Summarizes environmental or anthropogenic context influencing ARGs (e.g., aquaculture, urban runoff, sediment type)

Supplementary Table S2. Detailed summary of included mangrove studies and contextual references

Study	Year	Country / Region	Sample Type	Detection Method	ARGs Detected	ARG Abundance	Notes / Context	Reference
Jiang et al.	2021	Hainan, China	Sediment	qPCR	*tetA, tetM, sul1*	10^3–10^5 copies/g	Mangrove sediment near aquaculture	[11]
Palacios et al.	2021	Mexico	Water	Metagenomics	*mdt, acr*	10^2–10^4 copies/mL	Urban runoff impacted mangrove	[1]
Zhao et al.	2023	South China	Sediment	Metagenomics	*sul2, bla_CTX-M*	10^3–10^6 copies/g	Fine sediment, high organic content	[15]
Imchen et al.	2019	India	Sediment	16S + qPCR	*tetM, bla_TEM*	10^2–10^5 copies/g	Human-impacted mangrove	[14]
Zhang et al.	2009	China	Water & Sediment	Metagenomics	*tetA, sul1, bla_CTX-M, mdt*	10^3–10^6 copies/g	High prevalence of MGEs	[13]
Hendriksen et al.	2019	Global	Sediment	Metagenomics	Various ARGs	10^2–10^6 copies/g	Global sewage comparison; contextual reference	[4]
Sresung et al.	2024	Thailand	Water	qPCR	*sul1, sul2*	25–35% prevalence	Urban watershed; contextual	[6]
Yaikhan et al.	2024	Thailand	Water, Sediment	qPCR	*blaTEM, blaSHV, blaCTX-M*	20–30% prevalence	Enterobacteriaceae; contextual	[7]
Hinthong et al.	2024	Thailand	Sediment	PCR/qPCR	*blaTEM, blaCTX-M*	10^2–10^5 copies/g	Mangrove adjacent to aquaculture	[9]
Intahphuak et al.	2021	Thailand	Sediment	qPCR	*ermB, mefA*	5–15% prevalence	Human-associated sediment	[10]
Liu et al.	2023	China	Sediment	Metagenomics	*qnrS, qnrB*	5–10% prevalence	Coastal sediments; contextual	[2]
Cabello et al.	2013	Chile	Sediment	Culture-based + PCR	*mdtK, acrB*	15–25% prevalence	Aquaculture-influenced mangrove	[16]
Zhang et al.	2020	China	Water	qPCR	*qnrS, blaCTX-M*	10^2–10^5 copies/mL	Coastal urban influence; contextual	[5]

Notes:

- Mangrove primary studies are indicated in bold in the manuscript version of the table.

- Contextual/global references are included for comparison or discussion only; they are clearly marked in the Notes column.
- ARG abundance values are reported as in the original studies.
